# Exposure–Response Analysis of Osimertinib in Patients with Advanced Non-Small-Cell Lung Cancer

**DOI:** 10.3390/pharmaceutics14091844

**Published:** 2022-09-01

**Authors:** Thomas Rodier, Alicja Puszkiel, Evelina Cardoso, David Balakirouchenane, Céline Narjoz, Jennifer Arrondeau, Vincent Fallet, Nihel Khoudour, Monia Guidi, Michel Vidal, Xavier Declèves, Chantal Csajka, Jérôme Alexandre, Jacques Cadranel, Elizabeth Fabre, Marie Wislez, François Goldwasser, Benoit Blanchet

**Affiliations:** 1 Biologie du Médicament-Toxicologie, Hôpital Cochin, AP-HP, 75014 Paris, France; 2 Inserm UMR-S1144, Université Paris Cité, 75006 Paris, France; 3 School of Pharmaceutical Sciences, University of Geneva, 1205 Geneva, Switzerland; 4 Institute of Pharmaceutical Sciences of Western Switzerland, University of Geneva, 1205 Geneva, Switzerland; 5 Department of Clinical Biochemistry, Hôpital Européen Georges Pompidou, AP-HP, 75015 Paris, France; 6 Medical Oncology, Cochin-Port Royal, AP-HP, Université Paris Cité, 75006 Paris, France; 7 Department of Pulmonology and Thoracic Oncology, Tenon Hospital, Assistance Publique Hôpitaux de Paris and GRC 4, Theranoscan, Sorbonne Université, 75020 Paris, France; 8 Service of Clinical Pharmacology, Lausanne University Hospital and University of Lausanne, 1011 Lausanne, Switzerland; 9 Center for Research and Innovation in Clinical Pharmaceutical Sciences, Lausanne University Hospital and University of Lausanne, 1011 Lausanne, Switzerland; 10 UMR8038 CNRS, U1268 INSERM, Faculté de Pharmacie, PRES Sorbonne Paris Cité, CARPEM, Université Paris Cité, 75006 Paris, France; 11 ER2 et GRC THERANOSCAN, Faculté de Médecine Pierre et Marie Curie, Université Paris 6, 75005 Paris, France; 12 Department of Thoracic Oncology, Hôpital Européen Georges Pompidou, AP-HP, 75015 Paris, France; 13 Inserm, UMR-S970, Université Paris Cité, 75006 Paris, France; 14 Department of Thoracic Oncology, Cochin-Port Royal, AP-HP, Université Paris Cité, 75006 Paris, France

**Keywords:** osimertinib, non-small-cell lung cancer, pharmacokinetics, pharmacodynamics

## Abstract

High interindividual variability (IIV) of the clinical response to epidermal growth factor receptor (EGFR) inhibitors such as osimertinib in non-small-cell lung cancer (NSCLC) might be related to the IIV in plasma exposure. The aim of this study was to evaluate the exposure–response relationship for toxicity and efficacy of osimertinib in unselected patients with advanced EGFR-mutant NSCLC. This retrospective analysis included 87 patients treated with osimertinib. Exposure–toxicity analysis was performed in the entire cohort and survival analysis only in second-line patients (*n* = 45). No significant relationship between occurrence of dose-limiting toxicity and plasma exposure was observed in the entire cohort (*p* = 0.23, *n* = 86). The median overall survival (OS) was approximately two-fold shorter in the 4th quartile (Q4) of osimertinib trough plasma concentration (>235 ng/mL) than in the Q1–Q3 group (12.2 months [CI95% = 8.0–not reached (NR)] vs. 22.7 months [CI95% = 17.1–34.1]), but the difference was not statistically significant (*p* = 0.15). To refine this result, the exposure–survival relationship was explored in a cohort of 41 NSCLC patients treated with erlotinib. The Q4 erlotinib exposure group (>1728 ng/mL) exhibited a six-fold shorter median OS than the Q1–Q3 group (4.8 months [CI95% = 3.3-NR] vs. 22.8 months (CI95% = 10.6–37.4), *p* = 0.00011). These results suggest that high exposure to EGFR inhibitors might be related to worse survival in NSCLC patients.

## 1. Introduction

Activating mutations in the epidermal growth factor receptor (EGFR) are key drivers of non-small-cell lung cancer (NSCLC) in 10–15% of Caucasian patients and 50% of Asian patients [1,2]. Tyrosine kinase inhibitors (TKI) inhibit activity of common EGFR variants (L858R mutation and exon 19 deletion) by binding to the EGFR receptor at its adenosine triphosphate (ATP)-binding site. After initial activity of 9–13 months with first- (erlotinib, gefitinib) and second-generation EGFR-TKI (afatinib), resistance develops in 50–60% of cases because of the T790M mutation in exon 20 of the *EGFR* gene [3]. EGFR T790M mutation increases ATP-binding activity to EGFR, which results in inefficiency of first- and second-generation TKIs. Osimertinib, a third-generation EGFR-TKI, is an irreversible EGFR inhibitor developed to target the T790M mutation. Among patients with NSCLC harboring the EGFR T790M mutation who have been previously treated with first- or second-generation EGFR-TKI, a phase 3 trial (AURA 3) demonstrated striking efficacy of osimertinib compared to intravenous pemetrexed plus either carboplatin or cisplatine [4]. Osimertinib is indicated for the treatment of NSCLC patients with metastatic EGFR T790M-positive NSCLC resistant to first- or second-generation EGFR-TKIs [4]. Furthermore, osimertinib has recently shown clinical benefit as a first-line treatment for EGFR mutation-positive NSCLC compared with a standard of care including first-generation EGFR-TKIs (erlotinib or gefitinib) [5]. Since then, osimertinib has also been approved as the first-line treatment in NSCLC patients whose tumors have EGFR exon 19 deletions or exon 21 L858R mutations.

In EGFR T790M-positive NSCLC patients, the response rate with osimertinib is similar in the daily dose range of 20–240 mg, and no dose-limiting toxicities (DLT) were reported in [6]. High osimertinib doses (160–240 mg) can inhibit wild-type EGFR but might result in the development of severe adverse events [6]. The recommended dose of osimertinib is 80 mg once daily in NSCLC patients regardless of the line of treatment [7]. The most common adverse events are diarrhea, rash, nausea and loss of appetite. The pharmacokinetics (PK) of osimertinib exhibits a moderate to large interindividual variability (IIV) [8]. Osimertinib is metabolized by CYP3A4/5 into two pharmacologically active metabolites, AZ7550 and AZ5104, that circulate at approximately 10% of the plasma exposure of the parent compound [9]. Most recently, the contribution of CYP1A2 to osimertinib metabolism has been reported in murine models [10]. Finally, osimertinib is a substrate of the multidrug efflux transporters ABCB1 (*p*-glycoprotein) and ABCG2 (BCRP) [11].

Several studies reported a relationship between drug plasma exposure and the occurrence of severe toxicities of EGFR inhibitors such as erlotinib [12,13,14,15] and afatinib [15,16,17,18,19,20]. In contrast, the relationships between plasma drug exposure and efficacy are sparse and remain controversial [21]. Regarding osimertinib, a pharmacokinetic/pharmacodynamic (PK/PD) study was conducted in NSCLC patients (*n* = 748) and healthy volunteers (*n* = 32) included in clinical trials [8]. No statistically significant relationship was found between plasma drug exposure and efficacy, but the severity of toxicities such as rash, diarrhea and QTcF enhanced with increasing plasma osimertinib exposure. Real-world NSCLC patients present more comorbidities (elderly, hepatic dysfunction, sarcopenia) and have higher risk for drug–drug interactions than selected patients in the clinical trials. In this context, one can expect an increased IIV in osimertinib PK and higher rate of severe toxicity events in real-world patients. As far as we know, no PK/PD study for osimertinib using population approach has been conducted in a cohort of NSCLC in unselected patients. The aims of this study were to describe the steady-state PK profile of osimertinib using non-linear mixed effects modelling (population approach), then to investigate the exposure–response relationship for toxicity and efficacy in unselected patients with advanced EGFR T790M-positive NSCLC.

## 2. Methods

### 2.1. Study Population and Data Collection

The study was conducted in unselected adult patients with NSCLC and EGFR-activating mutations treated with osimertinib. Patients were consecutively included between October 2017 and April 2022. Patients were followed in the Cochin University Hospital (Paris, France), Georges Pompidou European Hospital (Paris, France) or Tenon Hospital (Paris, France). The recommended starting dose was 80 mg once daily. The starting dose was reduced in patients for whom a high risk of toxicity was identified at initial clinical assessment. Subsequently, doses could be adjusted based on efficacy and safety. Demographic and biological data, co-medications and clinical events (toxicity, disease progression or death) were retrospectively collected from medical records.

### 2.2. Ethics

This study was conducted in accordance with the 2008 Declaration of Helsinki and was approved by the local ethics committee in Oncology (CLEP number: AAA-2022–08024). All patients provided written informed consent for the collection of their medical and pharmacogenetic data.

### 2.3. Plasma Drug Assay

Plasma concentrations were determined at steady state (i.e., at least 10 days after start of treatment or dose modification) at any time over the dosing interval during routine follow-up visits to the outpatient clinic. Blood samples (5 mL) were collected in lithium heparin-containing tubes. After centrifugation, the plasma was separated and stored at −20 °C until analysis. Osimertinib plasma concentrations were assayed using a validated liquid chromatography method coupled with tandem mass spectrometry [22] in the Laboratory of Pharmacology in the Cochin University Hospital. The calibration was linear in the range of 5–1000 ng/mL. The lower limit of quantification (LLOQ) was 5 ng/mL. The mean inter- and intra-day precision (expressed as coefficient of variation, CV) were 7.9% and 8.1%, respectively. The accuracy of the method was ensured by participation in the TKI Proficiency Testing Scheme provided by the Group of Clinical Pharmacology in Oncology (GPCO, Unicancer, Villejuif, France).

### 2.4. Pharmacogenetic Analysis

Six single-nucleotide polymorphisms (SNPs) in five genes involved in osimertinib PK were selected based on a literature search (PharmGKB). Genomic DNA was extracted from plasma using the QiaAmp DNA mini kit (Qiagen, Courteboeuf, France) in accordance with the manufacturer’s recommendations. The following SNPs were identified using Taq Man^®^ Drug Metabolism Genotyping Assays (Applied Biosystems, Courtaboeuf, France): CYP3A5 6986 A>G (rs776746, CYP3A5*3, C__26201809_30), CYP3A4 c.522-191 C>T (rs35599367, CYP3A4*22, C_59013445_10), CYP1A2 c.-163 C>A (rs762551, CYP1A2*1F, C___8881221_40), ABCB1 c.3435 C>T (rs1045642, C___7586657_20) and ABCB1 c.2677 G>T/A (rs2032582, C_11711720D_40 and C_11711720C_30) and ABCG2 c.421 C>A (rs2231142, C__15854163_70).

### 2.5. Population Pharmacokinetic Analysis for Osimertinib

#### 2.5.1. Structural and Statistical Model

Population PK parameters were estimated by computing the maximum likelihood estimator of the parameters without any approximation of the model (no linearization) by maximum likelihood using the stochastic approximation expectation–maximization (SAEM) algorithm implemented in the Monolix Suite (version 2020R1, Lixoft^®^, Anthony, France). The model that best fits the osimertinib PK data was identified by using a stepwise procedure, comparing one- and two-compartment models with first- and/or zero-order absorption with and without absorption lag time and first-order elimination. The IIV in PK parameters was described using a log-normal distribution. Proportional and combined error models were tested to describe the residual unexplained variability. Plasma samples with concentrations below the LLOQ of the assay, drawn before the steady state was achieved, or with missing time after the last dose intake, were excluded from the analysis.

#### 2.5.2. Covariate Analysis

The effect of baseline covariates potentially influencing osimertinib PK was first explored graphically. The following baseline covariates were tested: age, body mass index (BMI), sex, smoking status, aspartate aminotransferase (AST), alanine aminotransferase (ALT), total bilirubin, C-reactive protein (CRP), lactate dehydrogenase (LDH) and alkaline phosphatase (ALP). Baseline co-medications that could potentially affect CYP3A4/5, CYP1A2, ABCB1 and ABCG2 activities as well as those affecting absorption (proton pump inhibitors, PPIs) were tested. Continuous covariates were tested using the following equation:

$$\theta =\theta_{1}\times {\left(\frac{cov}{mean\;cov}\right)}^{\theta_{2}}\times e^{\eta \theta }$$

where *θ* is the PK parameter, *θ*_1_ is the mean population estimate for this parameter, *cov* is the individual continuous covariate value, *mean cov* is the mean value of the covariate in the studied population, *θ*_2_ is the effect of covariate on the parameter and *η_θ_* is the random effect defining the IIV for θ. The missing values for continuous covariates were imputed with the population mean value.

Dichotomous covariates (coded as indicator variables 0 or 1) were generally tested with exponential equation:

$$\theta =\theta_{1}\times e^{\left(\theta_{2}\times cov\right)}\times e^{\eta \theta }$$

where θ is the PK parameter, $\theta_{1}$ is the mean population parameter estimate for the reference group (i.e., when *cov* = 0), $\theta_{2}$ is the effect of the covariate on the parameter (i.e., when *cov* = 1), *cov* is the categorical covariate and ηθ is the random effect defining the IIV for θ. Co-medications were coded as 0 if absent and 1 if present; smoking status was coded as 0 for non-smoker and 1 for ex-smoker or current smoker. Pharmacogenetic covariates were coded as 0 for wild-type homozygous genotype and 1 for heterozygous and homozygous mutant genotypes, except for the CYP3A5*3 genotype, which was coded as 0 for expressors (heterozygous mutant *1/*3 or homozygous wild-type genotypes *1/*1) and 1 for nonexpressors (homozygous mutant, *3/*3).

#### 2.5.3. Parameter Estimation and Model Selection

The log-likelihood ratio test, based on the reduction of the objective function value (∆OFV), was used to discriminate between hierarchical models. A decrease in OFV of at least 3.84 (*p* < 0.05) and an increase of at least 6.63 (*p* < 0.01) points were considered statistically significant for one additional parameter in the model-building process or forward insertion and backward deletion covariate steps, respectively. The validation of the final PK model was performed using a visual predictive check (VPC) based on 500 simulations of the original dataset.

#### 2.5.4. Osimertinib Individual PK Parameters

Osimertinib individual PK parameters (empirical Bayes estimates, EBEs) were obtained with the final PK model developed in this study. Steady-state trough concentrations (C_min,ss_) were estimated for each patient at each sampling occasion (which allowed taking into account dose modifications) based on EBEs. Area under the concentration–time curve over the dosing interval at steady state (AUC) was calculated for each patient using integration method according to the following equation:

$$AUC=\int_{0}^{t}C\left(t\right)\times dt$$

where *C* is individual osimertinib plasma concentration estimated by the final model using EBEs. For the exposure–toxicity analysis, a mean osimertinib C_min,ss_ or AUC from the first three months of treatment was calculated for patients who did not present any DLT. For the exposure–survival analysis, a mean of all available C_min,ss_ or AUC was calculated for each patient.

### 2.6. Pharmacokinetic Analysis for Erlotinib

The results of the exposure–response relationship for osimertinib led us to explore whether this result was specific to the drug or to the EGFR-TKI class. For this purpose, we investigated the exposure–survival relationship in a cohort of NSCLC patients treated with erlotinib. Patients were consecutively included between August 2010 and December 2017. The erlotinib cohort was previously described [23]. A subgroup of these patients for whom efficacy data were available was included in the current study to evaluate the exposure–response relationship for survival. Erlotinib individual PK parameters (EBEs) were obtained using population PK model developed in a cohort investigated by Cardoso et al. [23]. C_min,ss_ were estimated for each patient at each sampling occasion based on EBEs. A mean of all available C_min,ss_ was calculated for each patient and used in the survival analysis.

### 2.7. Clinical Endpoints

Regarding safety analysis for osimertinib, the onset of DLT was considered as the primary endpoint. A DLT was defined as any toxicity leading to dose reduction or temporary or permanent discontinuation of treatment.

Regarding efficacy (erlotinib and osimertinib) analysis, the primary endpoint was progression-free survival (PFS), defined as the time from treatment initiation to a documented progression event (either clinical or radiological) or death from any cause. The secondary endpoint was overall survival (OS), defined as the time from treatment initiation to death from any cause. Radiographic evidence of progression was defined according to a modified version of Response Evaluation Criteria in Solid Tumors (RECIST) v1.1.

### 2.8. Statistical Analysis

For descriptive analyses, qualitative variables were expressed as number (%) and quantitative variables as median [interquartile range]. The correlation between PK and DLT was evaluated in all NSCLC patients treated with osimertinib regardless of the line of treatment. Only patients with at least one osimertinib concentration available within the first three months of treatment were included in the analysis. Comparisons between groups (with or without DLT) were performed using the non-parametric Wilcoxon test for quantitative variables and the Fisher test for qualitative variables. The following variables were tested: sex, age, BMI, Eastern Cooperative Oncology Group Performance Status (ECOG PS: 0–1 versus ≥ 2), cerebral metastases, smoking status, genetic polymorphisms (CYP3A5, CYP3A4, CYP1A2, ABCB1 and ABCG2) and plasma drug exposure (AUC, C_min,ss_). The last C_min,ss_ or AUC before the onset of DLT in patients who experienced a DLT was compared with the mean C_min,ss_ or AUC from the first three months of treatment in patients who did not experience any DLT.

Exposure–survival analyses were conducted in the cohort of patients treated with osimertinib as a second-line treatment. Concerning erlotinib, the analysis was performed in first- or second-line treatment. Survival curves were obtained with Kaplan–Meier estimates and compared using a log-rank test. Cox proportional hazards models were used to identify clinical and biological variables associated with survival (PFS, OS). The following variables were tested in the osimertinib cohort: sex, age, BMI, ECOG PS (0–1 versus ≥ 2), cerebral metastases (presence versus absence), albumin, CRP, LDH level, PPI intake (no intake versus intake), smoking status (non-smoker versus ex-smoker and current smoker), pharmacogenetic covariates and mean plasma exposure (AUC, C_min,ss_) over the entire treatment period. For the erlotinib survival analysis, the covariates sex, ECOG PS (0–1 versus ≥ 2), albumin, age, CRP, smoking status (non-smoker versus ex-smoker and current smoker) and mean plasma exposure (C_min,ss_) over the entire treatment period were tested. In the case of non-normal distribution as evaluated by Shapiro–Wilk test, the covariates were included as log-transformed values. All variables with *p* < 0.05 in univariate analysis were included in the multivariable Cox model. Backward elimination strategy was used to delete variables that contribute the least until the final model. Death rate was defined as a binary covariate and was coded as 1 if death occurred within 12 or 24 months after treatment start. All the PK/PD analyses were performed using R program (version 4.0.3, http://www.r-project.org, accessed on 10 October 2020) with RStudio (version 1.3.1093).

## 3. Results

### 3.1. Patients

**Figure 1:**
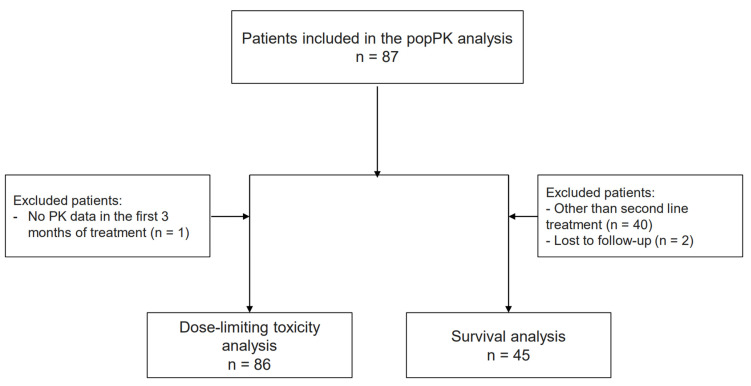
Osimertinib PK/PD study flowchart.

Demographic and biological characteristics of 87 patients included in the osimertinib cohort are summarized in Table 1. Osimertinib was administered at doses ranging from 40 mg to 160 mg once daily. The flowchart of the osimertinib cohort included in the PK/PD analysis is presented in Figure 1.

The erlotinib cohort included 41 NSCLC patients and their clinical and biological characteristics are summarized in Table 2. Erlotinib was administered at doses ranging from 25 mg to 400 mg once daily.

### 3.2. Pharmacogenetic Data

Variant allele frequencies were assessed in 86 patients (98.9%) from the osimertinib cohort and were in accordance with those observed in the overall Caucasian population (>75% of the population were Caucasians, Table 3) [24,25]. Genotypes were distributed according to the Hardy–Weinberg equilibrium except for the CYP3A5. However, these deviations were not observed when only Caucasian patients were considered.

### 3.3. Osimertinib Population PK Analysis

The population PK analysis included a total of 420 plasma osimertinib concentrations. A median of three samples per patient (range 1–28) was collected between 0.33 and 27 h (median = 18 h) after the last dose intake. The median time from treatment start to blood collection was 115 days (IQR: 40–281). Steady-state osimertinib concentration–time data were described by a one-compartment model with first-order absorption and elimination. Since few PK data were available in the absorption phase, first-order absorption rate constant (k_a_) was fixed to 0.24 h^−1^ according to a previously published model [8] to allow an adequate estimation of all PK parameters. A two-compartment model was not associated with a significant improvement (∆OFV = −1.58, *p* = 0.21). A proportional error model was used to describe the residual unexplained variability. The IIV was included on apparent clearance (CL/F) and apparent central volume of distribution (V/F) and was associated with acceptable RSE and shrinkage (Table 4).

**Figure 2:**
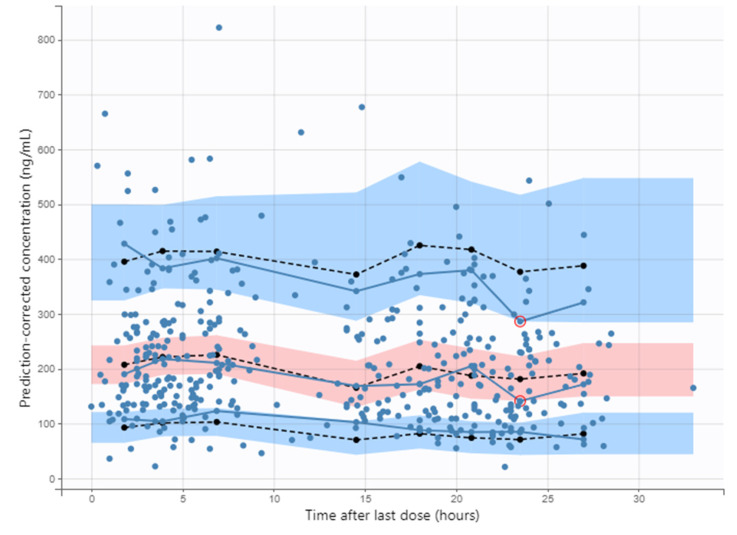
Prediction-corrected visual predictive check of osimertinib final PK model. Dashed lines represent the 5th, 50th and 95th percentiles of simulated concentrations, blue solid lines represent the 5th, 50th and 95th percentile of observed concentrations, shaded areas represent the 90% prediction intervals around the 5th, 50th and 95th percentiles of simulated data and blue points represent the observed data. Red circles represent outliers (i.e., when the empirical percentile is not comprised within the 90% prediction interval for that percentile).

The univariate covariate analysis identified a significant relationship between osimertinib CL/F and *ABCB1* c.3435C>T (ΔOFV = −3.87, *p* = 0.049), sex (ΔOFV = −5.15, *p* = 0.02), ethnicity (ΔOFV = −4.5, *p* = 0.034), *p*-gp inhibitors (ΔOFV = −8.53, *p* = 0.003) and CYP1A2 inducers (ΔOFV = −7.33, *p* = 0.007). Concomitant intake of PPI did not show any significant impact on CL/F (ΔOFV =−0.072, *p* = 0.79). The multivariate analysis identified only *ABCB1* c.3435C>T as an independent factor associated with CL/F (ΔOFV = 7.05, *p* = 0.008). However, the decrease in IIV of CL/F after inclusion of this covariate was marginal (decrease from 40% to 37%). Herein, *ABCB1* c.3435C>T was not included in the final model. The final model parameter estimates are presented in Table 4. The prediction-corrected VPC supports an adequate description of the observed osimertinib concentrations (Figure 2). Median osimertinib C_min,ss_ and AUC during the entire follow-up time was 200.5 ng/mL [146–235 ng/mL] and 5266 ng/mL.h [3950–6170 ng/mL.h], respectively.

### 3.4. Exposure–Toxicity Analysis for Osimertinib

Out of 86 patients (Figure 1), 13 patients (15.1%) experienced DLT (10 dose reductions to 40 mg/day, 3 definitive discontinuations) including diarrhea (*n* = 4), interstitial lung disease (*n* = 2), hepatotoxicity (*n* = 2), asthenia (*n* = 2), thrombopenia (*n* = 2), mucositis (*n* = 2) and cardiac failure (*n* = 1). The median time to DLT onset was 73 days [56–116 days]. The list of the observed DLT per patient in presented in Supplemental Table S1. In patients who presented a DLT, median AUC was 5786 ng/mL.h [5555–5794 ng/mL.h] compared with 5202 ng/mL.h [4112–6959 ng/mL.h] in patients without DLT (*p* = 0.23). Median C_min,ss_ in patients who presented a DLT was 217 ng/mL [199–287 ng/mL] versus 201 ng/mL [153–264 ng/mL] in patients who did not experience a DLT (*p* = 0.27). None of the tested biological variables including genotypes and PK parameters were identified as risk factors of DLT (Table 5).

### 3.5. Exposure–Survival Analysis for Osimertinib

**Figure 3:**
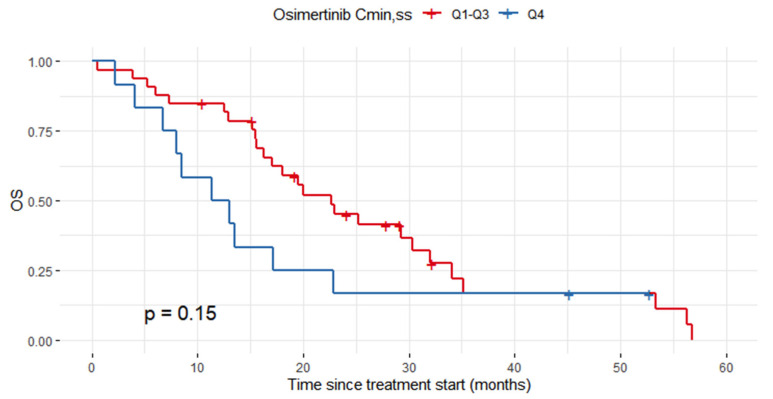
Overall survival (OS) in second-line osimertinib cohort according to osimertinib plasma exposure (the highest quartile of C_min,ss_ (Q4) versus all other quartiles (Q1–Q3)).

Forty-seven patients treated with osimertinib in the second line were included in the survival analysis (Figure 1). Two patients were lost to follow-up; the statistical analysis was therefore conducted in 45 patients. The median PFS and OS were 6.6 months (CI95% = 5.1–9.5) and 18.0 months (CI95% = 15.5–29.3), respectively. In multivariate analysis, logC_min,ss_ (hazard ratio, HR = 2.60, CI95% = 1.08–6.24) and smoking status (HR = 2.35, CI95% = 1.13–4.88) were independently associated with PFS (Table 6), whereas logC_min,ss_ (HR = 11.31, CI95% = 2.05–62.42) and CRP (HR = 1.03, CI95% = 1.01–1.06) were independently associated with OS. To better describe the influence of osimertinib exposure on OS, the cohort was dichotomized into patients in the highest quartile of C_min,ss_ (Q4) and all other quartiles (Q1–Q3). The median OS was approximately two-fold shorter in the Q4 group than in the Q1–Q3 group (12.2 months, CI95% = 8.0-not reached [NR] vs. 22.7 months, CI95% = 17.1–34.1), but the difference was not statistically significant (log-rank test *p* = 0.15, Figure 3). However, the death rate was statistically higher in the Q4 group at 1 year (50.0 vs. 17.9%, respectively; Fisher-exact *p* = 0.044) and 2 years (85.7 vs. 48.5%, respectively; Fisher-exact *p* = 0.047) after treatment start compared to the Q1–Q3 group.

### 3.6. Exposure–Survival Analysis for Erlotinib

**Figure 4:**
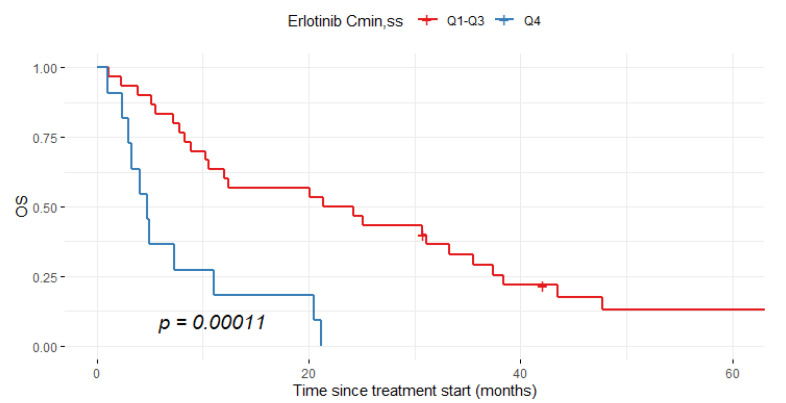
Overall survival (OS) in erlotinib cohort according to the highest quartile of Cmin,ss (Q4) and all other quartiles (Q1–Q3).

Given that a worse clinical benefit was observed in NSCLC patients overexposed to osimertinib, we decided to explore whether this result was specific to the drug or to the EGFR-TKI class. For this purpose, we investigated the exposure–survival relationship in a cohort of 41 NSCLC patients treated with erlotinib. The median erlotinib C_min,ss_ per patient was 1387 ng/mL [1009–1728 ng/mL]. The multivariate analysis identified C_min,ss_ as an independent risk factor of OS and PFS (Table 7). The cohort was dichotomized into patients in the highest quartile of C_min,ss_ (Q4) and all other quartiles (Q1–Q3). Figure 4 shows that the Q4 group exhibited a six-fold-shorter median OS than the Q1-Q3 group (4.8 months, CI95% = 3.3-NR vs. 22.8 months (CI95% = 10.6–37.4, respectively, log-rank test *p* = 0.00011).

## 4. Discussion

In the last fifteen years, EGFR-TKIs have become a gold standard in the treatment of EGFR-mutant NSCLC. However, 10–30% of patients develop acquired resistance within the first 6 months of treatment [26]. Response to TKIs might depend on plasma exposure [12,27], but data on the PK/PD relationship remain scarce for erlotinib [28] and osimertinib [8,27,28] in NSCLC patients. The present study reports that high plasma exposure to osimertinib or erlotinib (above 75th percentile) might result in worse PFS and OS in unselected NSCLC patients.

As far as we know, this is the first study to report osimertinib population PK analysis in unselected real-world patients. The steady-state osimertinib PK data were described by a one-compartment model with first-order absorption and elimination. The estimates of CL/F (13.7 L/h) and V/F (974 L) are coherent with a previous analysis (14.2 L/h and 986 L, respectively) [8]. The IIV in CL/F and V/F was 40% and 64%, respectively, which is consistent with previously reported values based on clinical trials’ data [8]. This study is also the first to investigate the impact of CYP3A5*3, CYP3A4*22, CYP1A2*1F, *ABCB1* c.3435 C>T, *ABCB1* c.2677 G>T/A and *ABCG2* c.421 C>A genetic polymorphisms on osimertinib PK. None of these covariates were significantly associated with osimertinib CL/F or V/F in our study. The contribution of CYP3A4/5 pathways in osimertinib metabolism remains limited, which can explain, in part, these results. In addition, the concomitant administration of CYP3A4, CYP1A2, ABCB1 and ABCG2 inhibitors or inducers did not have any significant impact on osimertinib PK in our cohort. The finding concerning CYP3A4 inhibitors is further supported by the fact that concomitant administration of itraconazole (strong CYP3A4 inhibitor) did not have any clinically relevant impact on osimertinib PK in a dedicated study [7]. Concerning CYP3A4 inducers, as described previously, rifampicin significantly decreases plasma exposure to osimertinib and requires a dose increase [29]. However, the impact of CYP3A4 inducers was not confirmed in our study most probably because of a low number of patients taking these co-medications. In addition, we did not observe any significant impact of PPI on osimertinib PK in our analysis, consistent with previous reports [30]. Medians of individual model-predicted AUC and C_min,ss_ were in accordance with those previously reported in clinical trials [8], which validates the use of the model-derived parameters as exposure metrics in our PK/PD analyses.

Osimertinib shows the most favorable safety profile among EGFR-TKIs [31]. In the present study, the frequency of dose reduction (11.6%) was close to that reported in the AURA 3 trial (16.5%) [4]. We did not find any association between increased plasma exposure and DLT occurrence. Brown et al. reported a relationship between increased steady-state AUC and a higher risk to develop adverse events such as skin rash, diarrhea and increase in cardiac QTc time [8]. However, the association between plasma exposure and DLT was not investigated. During the clinical drug development, no maximum tolerated dose could be defined over the 20–240 mg dose range [8]. Furthermore, a phase 2 study in 80 EGFR T790M-positive NSCLC patients treated for intracranial disease progression reported a manageable safety profile of a 160 mg/day dose with 17% of patients requiring dose adjustment [32]. Taken together, these results support the lack of association between DLT and plasma osimertinib exposure in our study.

The main finding of this report is that an increased osimertinib plasma exposure (Q4) is independently associated with shorter PFS and OS in patients in second-line treatment. Indeed, higher risk of death was observed in patients with C_min,ss_ > 75th percentile (Q4) compared to patients with C_min,ss_ < 75th percentile (Q1, Q2 and Q3). Similar results were previously observed in a PK/PD analysis based on 710 patients included in AURA trials [33]. In that study, shorter median PFS was observed in the highest exposure quartile (Q4) compared with the Q1–Q3 group (8.3 months [CI95% = 6.9–10.5] versus 11.2 months for all [CI95% = 9.7–12.7; 8.5–15.6 and 8.7–13.7, respectively]). The authors argued that Q4 included a larger number of patients with poor prognostic features (i.e., World Health Organization (WHO) performance status of 1 or 2 and lower baseline serum albumin) compared with the Q1–Q3 group, which could explain their worse survival. However, we investigated this hypothesis and did not find any significant difference in terms of ECOG PS, CRP level, albuminemia, smoking status or presence of brain metastases between the two subgroups of patients (Q1–Q3 versus Q4, Supplemental Table S2). Therefore, worse survival in patients with high plasma exposure could not be explained by their poorer prognostic factors in our study. Interestingly, a similar result for worse survival in the highest plasma exposure group (Q4) was observed in a cohort of EGFR-mutant NSCLC patients treated with erlotinib, suggesting a class effect for this PK/PD relationship. Indeed, we observed a higher risk of death in patients with erlotinib C_min,ss_ > 1728 ng/mL (75th percentile) compared with patients in the Q1–Q3 group of plasma exposure. Fukudo et al. previously reported that among NSCLC patients with EGFR mutations, those with the middle range of erlotinib concentrations (Q2–Q3, 848–1684 ng/mL) had better objective response rate (84%) than those with low (Q1, 67%) and high (Q3, 67%) concentrations [12]. However, the authors did not evaluate the relationship between plasma exposure and PFS nor with OS. Previous reports from the literature also suggest existence of an exposure–response relationship for efficacy of erlotinib. Indeed, Steedam et al. showed that the decrease in plasma erlotinib exposure over the treatment course could result in shorter PFS in NSCLC patients [27]. In addition, a C_min,ss_ threshold of > 500 ng/mL was proposed based on preclinical data [15,34], but it has never been confirmed in a clinical setting. Based on our findings, a middle-range exposure (Q1–Q3) could be targeted for both erlotinib and osimertinib in NSCLC patients. Even though our findings concerning worse survival in patients with the highest plasma exposure need confirmations in larger prospective cohorts, targeting middle-range exposure could limit the occurrence of severe toxicities and DLT, especially for erlotinib [12,14]. Furthermore, future studies should evaluate the PK/PD relationship for other EGFR-TKIs (gefitinib, afatinib) in order to confirm if our finding is a class effect for these drugs.

The unexpected result regarding worse survival in patients with high plasma exposure to osimertinib or erlotinib might be explained by the occurrence of tumor resistance to EGFR-TKIs through off-target mechanisms including amplification of MET and HER2 [35]. This phenomenon has been previously suggested for dabrafenib in BRAF-mutated metastatic melanoma patients [36]. However, the molecular analysis of tumor biopsies at disease progression was performed in a limited number of patients in our cohort; therefore, we could not answer this question. In the future, molecular evaluation of tumor biopsies at progression should be a critical factor to better characterize the relationship between resistance mechanisms to EGFR-TKIs and plasma drug exposure.

In the present study, baseline serum CRP level was identified as an independent risk factor of shorter OS for both osimertinib and erlotinib, consistent with previous reports. Indeed, a recent study showed a significant association between high baseline serum interleukin (IL)-6 level and shorter PFS in a cohort of 70 NSCLC patients treated with first- or second-line osimertinib [37]. IL-6 is also known as a driver of resistance to erlotinib [38,39], whereas high baseline serum CRP level is a negative prognostic factor of survival in NSCLC patients [40]. High IL-6 and CRP levels are also associated with inflammatory status and, as a result, decreased activity of CYP enzymes [41,42]. Rivory et al. documented a correlation between elevated CRP and reduced CYP3A4 activity in 40 patients with advanced cancer [43]. In this context, our PK/PD finding regarding worse OS in patients with high plasma exposure could be related to the inflammatory status occurring in some patients during the treatment course. However, our population PK analyses for osimertinib and erlotinib [23] failed to identify CRP level as significant covariables on CL/F. This consolidates the results of the multivariate survival analysis where both plasma drug exposure and CRP levels are significant predictors of OS. In addition, sarcopenia could explain, in part, the association between increased plasma drug exposure and worse survival. Indeed, sarcopenia can enhance the plasma drug exposure in cancer patients treated with TKIs [44,45]. In addition, although the data are contradictory, sarcopenia in NSCLC patients treated with EGFR-TKIs has been identified as a risk factor of poor prognosis and shorter survival [46,47,48]. In the present study, sarcopenia status was not available. In this context, it deserves to be investigated in the future PK/PD studies for EGFR-TKIs.

This study has several limitations. First, it included a low number of patients in both cohorts and the clinical data were retrospectively collected from medical records. Nevertheless, the median PFS and OS were coherent with those previously reported in clinical trials [4]. Furthermore, high rates of PFS and OS events for exposure–survival analysis in the two study cohorts (96% and 80% for osimertinib, 100% and 95% for erlotinib, respectively) are a strength of our retrospective analysis. Secondly, the PK analysis did not include active metabolites of osimertinib (AZ5104) and erlotinib (OSI-420). However, plasma exposure to these metabolites represents approximately 10–12% of that of the parent compound [49,50]; thus, their contribution to clinical activity seems limited. This is also supported by previous results of PK/PD analyses including these metabolites where no significant relationships between their plasma exposure and clinical response were identified [8,51,52]. Finally, the covariate step in PK analysis included baseline values of biologic variables including CRP level. Therefore, it might not reflect CRP levels during the treatment course. This could explain why a significant relationship between baseline CRP and osimertinib CL/F was not observed in our analysis. Time-varying CRP data could not be included in our analysis due to a high number of missing data in the medical records. Nevertheless, in the survival analysis, both plasma exposure and CRP are independently associated with OS, which excludes the possibility of correlation between the two variables.

## 5. Conclusions

In conclusion, this study shows that genetic polymorphisms in CYP3A4/5, CYP1A2 and efflux pumps (ABCB1 and ABCG2) do not have a significant impact on osimertinib PK. Regarding PK/PD analysis, DLT occurrence was not associated with increased plasma osimertinib exposure. Higher risk of death was observed in patients in the highest osimertinib plasma exposure quartile (Q4) and in patients with high baseline CRP levels. This result was confirmed in a cohort of unselected NSCLC patients treated with erlotinib, suggesting a class effect for this PK/PD relationship. Further investigations are warranted in order to confirm these findings and to clarify the mechanism of resistance to EGRF-TKIs.

## Figures and Tables

**Table 1:** Demographic and baseline characteristics of patients treated by osimertinib. Data are presented as median [25th–75th percentile] or number (%).

Characteristics	Median [25th–75th Percentile] or Number (%)		
	1st Line (*n* = 28)	2nd Line (*n* = 47)	≥3rd Line (*n* = 12)
Sex			
Female	16 (57.1)	34 (72.3)	10 (83.3)
Ethnicity			
Caucasian	21 (75.0)	31 (66.0)	12 (100)
African	2 (7.1)	11 (23.4)	0 (0)
Asian	5 (17.9)	5 (10.6)	0 (0)
Age (years)	63.0 [55.8–72.2]	68.0 [56.5–78.5]	69.5 [59.2–73.0]
BMI (kg/m^2^)	22.0 [19.6–24.4]	23.2 [21.0–26.0]	21.1 [17.9–23.9]
ECOG PS			
0–1	18 (64.3)	31 (66.0)	9 (75.0)
≥2	10 (35.7)	16 (34.0)	3 (25.0)
PPI intake			
Yes	9 (32.1)	10 (21.3)	2 (16.7)
CYP3A4 moderate and strong inhibitors	1 (3.6)	8 (17)	2 (16.7)
CYP3A4 moderate and strong inductors	0 (0)	1 (2.1)	1 (8.3)
Smoking status			
Non-smoker	14 (50)	35 (74.5)	8 (66.7)
Ex- and current smoker	14 (50)	12 (25.5)	4 (33.3)
Starting dose (mg/day)			
40	0 (0)	4 (8.5)	0 (0)
80	26 (92.9)	43 (91.5)	11 (91.7)
160	2 (7.1)	0 (0)	1 (8.3)
Cerebral metastases			
Yes	12 (42.9)	23 (48.9)	7 (58.3)
Number of metastatic sites			
≥3	8 (28.6)	15 (31.9)	7 (58.3)
Histological tumor type			
NSCLC adenocarcinoma	27 (96.4)	45 (95.7)	12 (100)
NSCLC NOS	1 (3.6)	2 (4.3)	0 (0.0)
Type of EGFR mutation			
Exon 18	2 (7.1)	3 (6.4)	0 (0)
Exon 19	14 (50)	30 (63.8)	6 (50)
Exon 20	3	27	6
Exon 21	12 (42.9)	14 (29.8)	6 (50)
ALT (UI/L)	24.0 [14.5–43.0]	26.0 [17.5–33.0]	18.0 [17.5–33.5]
AST (UI/L)	22.0 [18.0–30.5]	26.0 [22.0–31.5]	21.0 [18.0–27.5]
Total bilirubin (µmol/L)	6.9 [6.0–8.1]	6.0 [4.1–7.4]	6.0 [5.5–11.5]
Albumin (g/L)	40.0 [35.5–42.8]	38.0 [36.0–40.0]	41.0 [39.5–42.5]
CRP (mg/L)	6.3 [2.1–23.0]	3.5 [2.1–19.3]	2. 5 [1.3–2.9]
Creatinine (µmol/L)	64.5 [58.0–75.5]	82.0 [70.0–94.0]	74.5 [61.0–82.5]
LDH (UI/L)	258 [196–278]	230 [188–271]	253 [235–271]

ALT, alanine amino transferase; AST, aspartate amino transferase; BMI, body mass index; CRP, C-reactive protein; ECOG PS, Eastern Cooperative Oncology Group Performance Status; EGFR, Epidermal Growth Factor Receptor; LDH, lactate dehydrogenase; NOS, not otherwise specified; NSCLC, non-small-cell lung cancer; PPI, proton pump inhibitors.

**Table 2:** Demographic and baseline characteristics of patients treated by erlotinib. Data are presented as median [25th–75th percentile] or number (%).

Characteristics	Median [25th–75th Percentile] or Number (%)
Sex	
Female	25 (61)
Ethnicity	
Caucasian	32 (78)
Other	9 (22)
Age (years)	73.3 [60.1–81.1]
BMI (kg/m^2^)	61.0 [54.0–72.0]
ECOG PS	
0–1	30 (73.2)
≥2	11 (26.8)
Smoking status	
Non-smoker	26 (63.4)
Ex-smoker and current smoker	15 (36.6)
Treatment line	
1st line	25 (61.0)
2nd line	16 (39.0)
Starting dose (mg/day)	
150	37 (90.2)
100	3 (7.3)
75	1 (2.5)
C_min,ss_ (ng/mL)	1387 [1009–1728]
ALT (UI/L)	21.0 [15.0–35.5]
AST (U/L)	24.0 [20.0–34.8]
Total bilirubin (µmol/L)	12.0 [8.0–14.5]
Albumin (g/L)	36.0 [32.2–39.0]
CRP (mg/L)	5.5 [5.0–22.2]
Creatinine (µmol/L)	75 [68.0–94.0]

ALT, alanine amino transferase; AST, aspartate amino transferase; BMI, body mass index; CRP, C-reactive protein; ECOG PS, Eastern Cooperative Oncology Group Performance Status.

**Table 3:** Distribution of the studied genotypes in the osimertinib cohort.

Gene	Allele	Rs Number	Annotation	wt/wt, *n* (%)	wt/m, *n* (%)	m/m, *n* (%)	Minor Allele Frequency	Missing Genotype Data, *n* (%)	HWE *p*-Value
CYP3A5	6986 A>G	rs776746	CYP3A5*3	12 (14)	15 (17)	59 (69)	0.14	1 (1.2)	<0.001 ^a^
CYP3A4	c.522–191 C>T	rs35599367	CYP3A4*22	79 (92)	7 (8)	0 (0)	0.06	1 (1.2)	0.69
CYP1A2	c.-163 C>A	rs762551	CYP1A2*F	11 (13)	39 (45)	36 (42)	0.37	1 (1.2)	0.93
ABCB1	3435 C>T	rs1045642	-	28 (33)	46 (53)	12 (14)	0.48	1 (1.2)	0.32
ABCB1	2677 G>T/A	rs2032582	-	33 (38)	44 (51)	9 (11)	0.41	1 (1.2)	0.31
ABCG2	c.421 C>A	rs2231142	-	68 (82)	14 (17)	1 (1)	0.08	4 (4.6)	0.77

HWE, Hardy–Weinberg Equilibrium; m, mutant allele; SNP, single-nucleotide polymorphism; wt, wild-type allele. ^a^ the less common allele for CYP3A5 was the A-allele (wild-type allele) in our mostly Caucasian population.

**Table 4:** Parameter estimates of the final osimertinib pharmacokinetic model.

Parameter	Mean Estimate	RSE (%)
CL/F (L/h)	13.7	7.2
V/F (L)	974	17.5
k_a_ (1/h)	0.24 (fixed)	-
IIV_CL/F_	0.40	11.7
IIV_V/F_	0.64	30.4
Proportional residual variability	0.35	4.2

CL/F, apparent clearance; IIV, interindividual variability, k_a_, first-order absorption rate constant; RSE, relative standard error; V/F, apparent distribution volume.

**Table 5:** Risk factors for dose-limiting toxicity in EGFR-mutated NSCLC patients treated with osimertinib. Data are presented as median [25th–75th percentile] or number (%).

Parameters	DLT (*n* = 13)	No DLT (*n* = 73)	*p*-Value
AUC (ng/mL.h)	5786 [5555–7494]	5202 [4112–6959]	0.23
C_min,ss_ (ng/mL)	217 [199–287]	201 [153–264]	0.27
Age (years)	68.0 [62.0–72.0]	66.0 [54.0–74.0]	0.41
BMI (kg/m^2^)	20.2 [19.4–23.1]	23.0 [20.6–26.0]	0.15
Sex			0.21
Male	2 (15.4%)	25 (34.2%)	
Female	11 (84.6%)	48 (65.8%)	
Presence of cerebral metastases	7 (53.8%)	34 (46.6%)	0.75
ECOG PS			0.003
0–1	13 (100%)	44 (60.3%)	
≥2	0 (0.00%)	29 (39.7%)	
Concomitant PPI	3 (23.1%)	18 (24.7%)	1.0
Smoking status			0.76
ex-smoker and current smoker	5 (38.5%)	25 (34.2%)	
non-smoker	8 (61.5%)	48 (65.8%)	
CYP3A4*22			0.58
wt/wt	12 (100%)	66 (91.7%)	
wt/m, m/m	0 (0.00%)	6 (8.33%)	
CYP3A5*3			0.54
wt/wt, wt/m	3 (23.1%)	24 (33.3%)	
m/m	10 (76.9%)	48 (66.7%)	
CYP1A2*F			0.67
wt/wt	2 (15.4%)	9 (12.5%)	
wt/m, m/m	11 (84.6%)	63 (87.5%)	
ABCG2 c.421 C>1			0.68
wt/wt	7 (77.8%)	57 (81.4%)	
wt/m, m/m	2 (22.2%)	13 (18.6%)	
ABCB1 3435 C>T			0.53
wt/wt	3 (23.1%)	25 (34.7%)	
wt/m, m/m	10 (76.9%)	47 (65.3%)	
ABCB1 2677 G>T/A			0.07
wt/wt	2 (15.4%)	31 (43.1%)	
wt/m, m/m	11 (84.6%)	41 (56.9%)	

AUC, area under the plasma concentration over interval administration; BMI, body mass index; C_min,ss_, trough concentration at steady state; DLT, dose-limiting toxicity; ECOG PS, Eastern Cooperative Oncology Group Performance Status; LDH, lactate dehydrogenase.

**Table 6:** Univariate and multivariate Cox proportional hazards models for risk factors of death and disease progression in patients treated in second line with osimertinib.

Univariate Model	Risk of Death		Risk of Progression	
	HR (CI95%)	*p*-Value	HR (CI95%)	*p*-Value
Age	1.00 (0.98–1.03)	0.884	0.99 (0.96–1.01)	0.368
Sex (Female vs. male)	0.99 (0.46–2.13)	0.989	0.67 (0.34–1.33)	0.255
ECOG PS ≥ 2	2.00 (0.97–4.09)	0.059	1.28 (0.67–2.44)	0.448
Presence of cerebral metastases	1.72 (0.86–3.44)	0.122	2.01 (10.6–3.80)	0.031
Albumin	0.99 (0.94–1.04)	0.575	1.01 (0.96–1.07)	0.625
CRP	1.03 (1.00–1.05)	0.015	1.04 (1.01–1.06)	0.004
LDH	1.00 (0.99–1.01)	0.976	1.00 (0.99–1.01)	0.728
CRP ≥ 10 mg/L	2.27 (0.77–6.70)	0.139	2.14 (0.73–6.32)	0.168
LDH ≥ 200 UI/L	0.70 (0.22–2.22)	0.543	0.69 (0.22–2.13)	0.519
Concomitant PPI	2.18 (0.94–5.08)	0.069	2.24 (1.05–4.79)	0.038
Smoking status (ex-smoker and current smoker vs. non-smoker)	1.47 (0.71–3.03)	0.300	2.09 (1.03–4.26)	0.041
CYP3A4*22 (wt/m, m/m vs. wt/wt)	1.21 (0.37–4.00)	0.757	0.71 (0.22–2.32)	0.567
CYP3A5*3 (m/m vs. wt/wt, wt/m)	0.63 (0.31–1.27)	0.199	0.88 (0.46–1.67)	0.687
CYP1A2*1F (wt/m, m/m vs. wt/wt)	0.78 (0.34–1.79)	0.551	0.52 (0.24–1.11)	0.092
ABCG2 c.421 C>A (wt/m, m/m vs. wt/wt)	0.51 (0.19–1.33)	0.169	0.58 (0.26–1.28)	0.180
ABCB1 3435 C>T (wt/m, m/m vs. wt/wt)	0.72 (0.36–1.46)	0.365	0.58 (0.30–1.12)	0.104
ABCB1 2677 G>T/A (wt/m, m/m vs. wt/wt)	0.53 (0.26–1.05)	0.068	0.69 (0.37–1.32)	0.264
Log AUC	2.97 (0.89–9.85)	0.031	2.25 (0.98–5.14)	0.055
Log C_min,ss_	3.01 (1.12–8.14)	0.030	2.20 (0.98–4.96)	0.056
**Multivariate Models**	**HR (CI95%)**	***p*-Value**	**HR (CI95%)**	***p*-Value**
Log AUC	11.61 (1.98–68.13)	0.007	2.73 (1.11–6.70)	0.029
CRP	1.03 (1.01–1.06)	0.008		
Smoking status (ex-smoker and current smoker vs. non-smoker)			2.41 (1.16–5.03)	0.019
Log C_min,ss_	11.31 (2.05–62.42)	0.005	2.60 (1.08–6.24)	0.033
CRP	1.03 (1.01–1.06)	0.009		
Smoking status (ex-smoker and current smoker vs. non-smoker)			2.35 (1.13–4.88)	0.022

ALP, alkaline phosphatase; ALT, alanine amino transferase; AST, aspartate amino transferase; AUC, area under the plasma concentration over interval administration; BMI, body mass index; CI95%, 95% confidence interval; C_min,ss_, trough concentration at steady state; CRP, C-reactive protein, ECOG PS, Eastern Cooperative Oncology Group Performance Status; LDH, lactate dehydrogenase; PPI, proton pump inhibitors.

**Table 7:** Univariate and multivariate Cox proportional hazards models for risk factors of death and disease progression in erlotinib cohort.

Univariate Model	Risk of Death		Risk of Progression	
	HR (CI95%)	*p*-Value	HR (CI95%)	*p*-Value
Second vs. first-line treatment	1.02 (0.53–1.96)	0.951	1.23 (0.66–2.31)	0.512
Sex (female vs. male)	0.73 (0.38–1.43)	0.363	0.70 (0.37–1.32)	0.269
ECOG PS ≥ 2	2.50 (1.17–5.34)	0.018	1.90 (0.94–3.84)	0.075
Albumin	0.93 (0.85–1.01)	0.084	0.93 (0.85–1.02)	0.115
CRP	1.01 (1.00–1.02)	0.012	1.02 (1.01–1.03)	0.004
Age	0.97 (0.95–1.00)	0.038	0.98 (0.96–1.00)	0.046
Age ≥ 50	0.13 (0.03–0.48)	0.002	0.25 (0.07–0.87)	0.029
Smoking status (ex-smoker and current smoker vs. non-smoker)	2.18 (1.12–4.24)	0.022	2.43 (1.29–4.58)	0.006
C_min,ss_/100	1.14 (1.05–1.24)	0.002	1.11 (1.03–1.19)	0.006
Multivariate Models	HR (CI95%)	*p*-Value	HR (CI95%)	*p*-Value
ECOG PS ≥ 2	2.84 (1.29–6.24)	0.009		
Age	0.97 (0.95–1.00)	0.041	0.97 (0.95–1.00)	0.031
Smoking status (ex-smoker and current smoker vs. non-smoker)	2.20 (1.09–4.46)	0.028	2.23 (1.14–4.37)	0.019
C_min,ss_/100	1.17 (1.08–1.28)	<0.001	1.16 (1.08–1.26)	<0.001
ECOG PS ≥ 2	2.97 (1.32–1.30)	<0.001		
Age ≥ 50	0.08 (0.02–0.34)	0.001	0.21 (0.06–0.74)	0.015
Smoking status (ex-smoker and current smoker vs. non-smoker)	2.29 (1.12–4.69)	0.023	2.41 (1.22–4.76)	0.011
C_min,ss_/100	1.19 (1.08–1.30)	<0.001	1.15 (1.07–1.25)	<0.001

CI95%, 95% confidence interval; C_min,ss_, steady-state trough concentration; CRP, C-reactive protein; ECOG PS, Eastern Cooperative Oncology Group Performance Status; HR, hazard ratio.

## Supplementary Materials

The following supporting information can be downloaded at: https://www.mdpi.com/article/10.3390/pharmaceutics14091844/s1. Supplemental Table S1. Details of the dose-limiting toxicities (*n* = 13 patients) observed in the osimertinib cohort. Supplemental Table S2 Distribution of covariates included in osimertinib and erlotinib survival analysis according to the highest quartile of C_min,ss_ (Q4) and all other quartiles (Q1–Q3). Data are presented as median [25th–75th percentile] or number (%). Supplemental Figure S1. Observed vs. individual predicted osimertinib concentrations obtained with the final PK model. Supplemental Figure S2. Goodness-of-fit plots of the final osimertinib PK model. IWRES individual weighted residuals, NPDE-normalized prediction distribution errors.
