# Supplementary material for: Exposure–Response Analysis of Osimertinib in Patients with Advanced Non-Small-Cell Lung Cancer
Source: Pharmaceutics. 2022 Sep 1;14(9):1844. doi: 10.3390/pharmaceutics14091844 (PMC9504753; doi:10.3390/pharmaceutics14091844)

Supplemental Table S1. Details of the dose-limiting toxicities (n = 13 patients) observed in the osimertinib cohort.

**ID**	**Type of Dose-limiting toxicity**
28	Hepatotoxicity
40	Diarrhea
41	Asthenia
42	Asthenia
50	Mucositis
55	Interstitial lung disease
59	Hepatotoxicity
61	Diarrhea
64	Cardiac failure
73	Diarrhea
76	Thrombopenia and diarrhea
77	Interstitial lung disease
79	Thrombopenia and mucositis

Supplemental Table S2. Distribution of covariates included in osimertinib and erlotinib survival analysis according to the highest quartile of $C_{min,ss}$ (Q4) and all other quartiles (Q1–Q3). Data are presented as median [25th-75th percentile] or number (%).

	Osimertinib			Erlotinib		
	Cmin,ss Q1-Q3 (n = 33)	Cmin,ss Q4 (n = 12)	p-value	Cmin,ss Q1-Q3 (n = 30)	Cmin,ss Q4 (n = 11)	p-value
**Age > 65 years old**	25 (75)	8 (67)	1	20 (67)	7 (64)	1
**ECOG PS**						
**0-1**	24 (73)	6 (50)	0.17	7 (23)	7 (64)	0.44
**≥2**	9 (27)	6 (50)		23 (77)	4 (36)	
**Presence of cerebral metastasis**	13 (40)	8 (67)	0.17	NA	NA	NA
**CRP (mg/L)**	3.5 [1.5-7.0]	12.90 [2.6-35.3]	0.63	6 [5.0-21.3]	5.5 [5.0-20.5]	1
**Albumin (g/L)**	38.0 [37.5-40.0]	35.5 [23.8-40.3]	0.80	35.5 [32.0-38.0]	37.5 [34.5-40.75]	0.30
**ASAT (UI/L)**	27.0 [22.0-31.3]	24.0 [21.3-36.5]	0.71	24.0 [19.8-36.5]	24.0 [20.0-33.0]	0.95
**ECOG PS**						
**0-1**	24 (73)	6 (50)	0.17	7 (23)	7 (64)	0.44
**≥2**	9 (27)	6 (50)		23 (77)	4 (36)	
**Smoking status**						
**Non-smoker**	10 (30)	12 (83)	0.47	20 (67)	6 (55)	0.49
**Ex- and current smoker**	23 (70)	2 (17)		10 (33)	5 (45)	

ASAT, aspartate amino transferase; $C_{min,ss}$ steady state trough concentration; CRP, C-reactive protein; ECOG PS, Eastern Cooperative Oncology Group Performance Status; NA, not assessed

Supplemental Figure S1. Observed vs individual predicted osimertinib concentrations obtained with the final PK model.

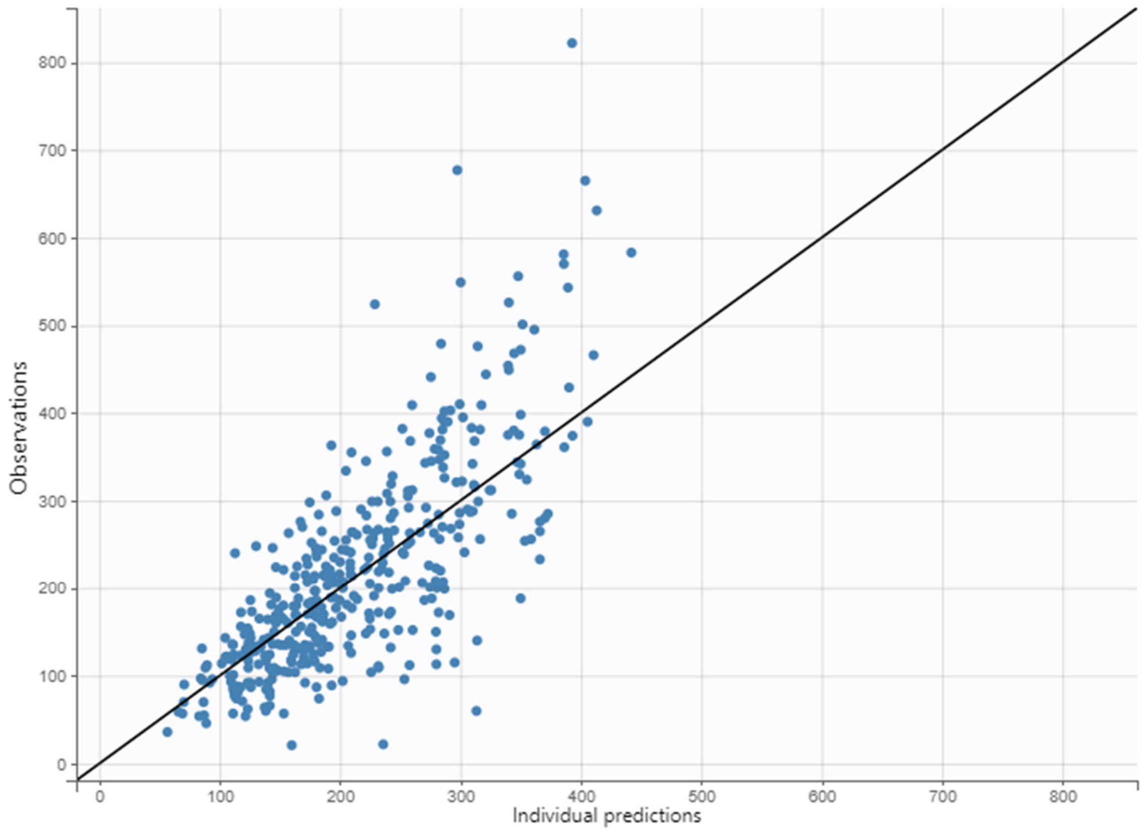

Supplemental Figure S2. Goodness of fit plots of the final osimertinib PK model. *IWRES* individual weighted residuals, *NPDE* normalised prediction distribution errors.

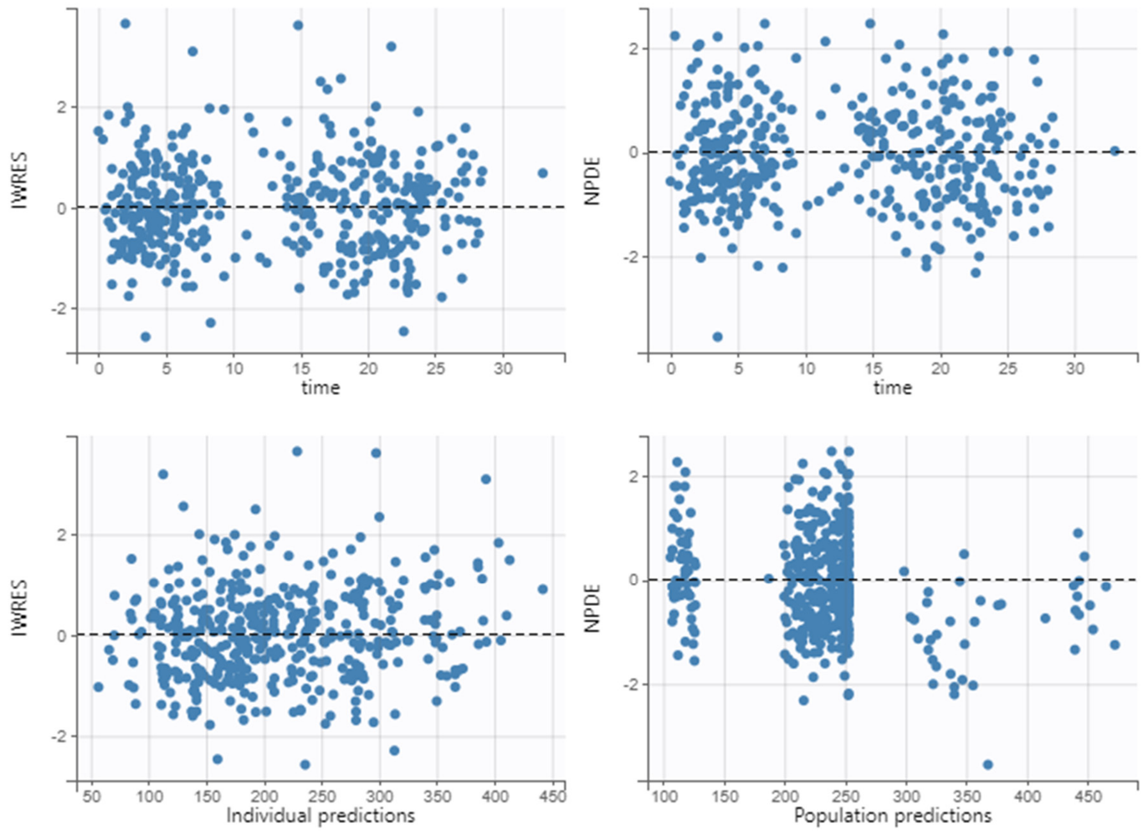

Supplement: Supplementary file 1 [file pharmaceutics-14-01844-s001.zip › pharmaceutics-1838815-supplementary.pdf]
